# Dental implant placement is a possible risk factor for the development of multiple cracks in non-endodontically treated teeth

**DOI:** 10.1038/s41598-020-65408-z

**Published:** 2020-05-22

**Authors:** Eyal Rosen, Yael Volmark, Ilan Beitlitum, Joseph Nissan, Carlos E. Nemcovsky, Igor Tsesis

**Affiliations:** 1 Department of Endodontology, Maurice and Gabriela Goldschleger School of Dental Medicine, Tel Aviv University, Tel Aviv, Israel; 2 Tel Aviv University Center for Nanoscience and Nanotechnology, Tel Aviv, Israel; 3 Department of Prosthodontics, Hebrew University - Hadassah, School of Dental Medicine, Jerusalem, Israel; 4 Department of Periodontology and Implant Dentistry, The Maurice and Gabriela Goldschleger School of Dental Medicine, Tel Aviv University, Tel Aviv, Israel; 5 Oral-Rehabilitation & Implant-Prosthodontics, Rabin Medical-Center, Belinson Hospital, Petah-Tikva, Israel; 6 Department of Oral-Rehabilitation, Maurice and Gabriela Goldschleger School of Dental Medicine, Tel Aviv University, Tel Aviv, Israel

**Keywords:** Dental diseases, Oral diseases, Diseases, Health care, Dentistry, Diagnosis, Prognosis

## Abstract

The objective of this study was to evaluate potential risk factors, including the placement of dental implants, for the development of tooth cracks. A series of 212-patients, who were referred for endodontic treatment, were retrospectively screened, of which 72 (34%) patients had been diagnosed with 80-cracked teeth confirmed with an operating microscope. These patients had an average age of 53-years and were equally distributed between genders. Forty-one percent of the cracked teeth were diagnosed after the placement of dental implants, with an average of 3-implants per patient. Seventy percent of the cracks were diagnosed more than 1-year after implant loading. Implant placement was associated with higher odds of having multiple cracks (OR = 9.78, CI:2.320, 41.216)(p < 0.05). The proportion of cracked premolars was relatively high (30%), and most cracked teeth (79%) were vital and with a normal periapical diagnosis (86%). Most cracked teeth (71%) had an amalgam restoration, and teeth restored with amalgam were at a higher risk of having multiple cracks (p < 0.05). Clinicians should be aware of a common profile of endodontic patients with multiple cracks in a non-endodontically treated premolar, restored with an amalgam restoration, which was diagnosed with the cracks more than 1-year after reconstruction utilizing multiple implants.

## Introduction

Excessive occlusal loads may lead to ensuing tooth fractures^1,2^. Longitudinal tooth fractures include two sub-types: fractures that initiate from the root (vertical root fracture, VRF)^3^, and fractures initiating from the crown and extending apically (cracked teeth)^3–5^. There are many potential risk factors for a cracked tooth that can be categorized into two sub-categories: natural origins (such as parafunctional habits, specific tooth anatomies, or certain patient ages) or iatrogenic origins (such as restorative procedures that caused excessive occlusal loads)^6,7^. Although the precise occlusal relationship between dental implants and adjacent natural teeth is not fully clarified^2,8–11^, recently it has been claimed that the placement of dental implants may be a potential risk factor for the development of tooth cracks, either in endodontically^3^ or in non-endodontically^12^ treated teeth. A systematic review of the literature published in 2016^3^ confirmed that this potential complication had not been reported prior to that review. Another recent study^12^ presented a series of 18 cases of non-endodontically treated teeth that were diagnosed with cracks after the reconstruction of an implant-supported rehabilitation, and proposed that “the most common patient profile would be a woman over 50 years old, having a cracked mandibular premolar tooth, which was diagnosed more than 1 year after reconstruction based on multiple implants”^12^. Nevertheless, due to the relatively small number of cases that were presented in these previous studies, the influence of potential risk factors on the development of tooth cracks following the placement of dental implants is yet unclear.

The objective of this study was to evaluate potential risk factors for the development of cracked teeth, including the placement of dental implants, based on a retrospective evaluation of patients that were referred for endodontic treatment.

## Methods

### Data collection and analysis

The study was approved by the Tel-Aviv University Ethics Committee on August 2, 2016, and all methods were performed in accordance with the relevant guidelines and regulations. Medical files of all patients who were referred to an endodontics clinic between January 2015 and June 2017 were retrospectively screened for the presence of cracks. All teeth were examined by the same endodontic specialist. A cracked tooth was defined as a tooth diagnosed with complete or incomplete tooth cracks initiating from the crown and extending apically^3–5^. The presence of cracks had been confirmed by visual examination using magnification and illumination with a dental operating microscope (Kaps 1400; Karl Kaps GmbH & Co. KG, Wetzlar, Germany). The coronal restoration had been removed for observation and the tooth had been stained using methylene blue dye (Methylene Blue; Dudley Chemical Corp, New Jersey, USA) when indicated^5^. The teeth were selected for further data analysis according to the inclusion and exclusion criteria.

### Inclusion criteria

1. Teeth diagnosed with a complete or incomplete crack initiating from the crown and extending apically, based on a documented clinical and radiographic evaluation^3–5^ (Digora Classic Phosphor plate system; Soredex Orion Corporation, Helsinki, Finland), and confirmed by visual examination using magnification and illumination with a dental operating microscope^5^.
2. the presence of occlusal contacts between the cracked tooth and the opposing tooth.

Teeth without confirmed cracks, endodontically treated teeth, and teeth diagnosed with VRF or craze lines^5,13^ were excluded from the analysis.

The following data were recorded for each patient based on the patient’s medical records and relevant radiographic examinations: the patient’s age and gender, the number and location of all dental implants that were placed before the diagnosis of the crack^3^, and the time from implant loading to the diagnosis of the crack. The cracked tooth type was categorized as a maxillary or mandibular anterior, premolar or molar. The pulp diagnosis was categorized as “vital”, when the tooth was diagnosed with either a normal pulp or pulpitis, or “non-vital”, when the pulp was necrotic^14^. The periapical diagnosis was categorized as “normal” or “with periapical pathology”^14^. The direction of the cracks was defined as mesio-distal (M-D), bucco-lingual (B-L), or both M-D and B-L^5^. The number of cracks was described as either single or multiple. The presence of adjacent teeth was recorded as “present” when both adjacent teeth were present, or “missing” when one or both of the adjacent teeth were missing at the time of crack diagnosis. The presence and the type of a coronal restoration was described as full coverage, amalgam, composite or none^5^. The extension of the coronal restoration was categorized as either a full coverage restoration, an extensive restoration (when the restoration covered two or more of the tooth crown aspects, but not the whole crown), a minor restoration (when the restoration covered one aspect of the tooth crown), or no restoration^5^. The presence of caries was recorded as “YES” or “NO”, while the presence of a deep periodontal pocket was described as “YES”, when probing depths >4 mm^1,5^.

### Statistical evaluation

The results were statistically analyzed using SPSS software version 22 (SPSS Inc, Chicago, IL). In the statistical evaluation of the results the numerical variables were described by average and standard deviation. The nominal variables were described by prevalence and percentage. The connections between parameters were tested with the Fisher’s exact test and the significance of the result got a BH correction. The statistical significance value was set to p < 0.05 for all statistical tests.

### Ethical approval

The study was approved by the Tel-Aviv University Ethics Committee and all methods were performed in accordance with the relevant guidelines and regulations.

### Informed consent

Informed consent was obtained from all individual participants included in the study.

## Results

A series of 212 patients were referred for endodontic treatment of 285 teeth during the time of data collection, of which 80 teeth of 72 patients complied with the inclusion criteria. Thus, the prevalence of a cracked tooth was 28% (80/285), diagnosed in 34% (72/212) of the patients that were examined during the study period. In cases where the evaluated parameters were not available from the patients’ medical records, the results were presented as a percentage from the number of cases in which that parameter was available.

Table 1 presents the characteristics of the cases diagnosed with cracked teeth. There were 36 (50%) women and 36 (50%) men between the ages of 29 and 76 years (average age 53 years) included in the analysis. Of these patients, 39 (54%) were over 50 years of age. Out of the 80 diagnosed cracked teeth, in 33 (41%) teeth the cracks were diagnosed after the placement of dental implants^3^. Among the patients with implants, in 9 (27.3%) patients, a single implant was present, in 9 (27.3%) patients two implants were placed, and in 15 (45.5%) patients three or more implants were placed (average 3 implants per patient). In 9 patients (27.3%) the implants were located adjacent to the cracked teeth, and in 17 (51.5%) patients the implants were located at opposing or contralateral sides, or at multiple sites in the mouth (n = 7, 21.2%). In 10 teeth (30.3%) the time from implant loading to the diagnosis of the crack was shorter than 1 year, in 12 (36.4%) teeth it was between 1 and 3 years, and in 11 (33.3%) teeth it was longer than 3 years.

**Table 1:** Characteristics of cases diagnosed with cracked teeth.

Parameter		Number of cases^7^	Percent^7^ (%)
Implant amount	Single	9	27.3
	Two	9	27.3
	Three or more	15	45.5
Implant location in relation to the cracked tooth	Adjacent	9	27.3
	Non adjacent	17	51.5
	Multiple sites	7	21.2
Time from implant loading to crack diagnosis	Less than 1 year	10	30.3
	Between 1 to 3 years	12	36.4
	More than 3 years	11	33.3
Gender	Women	36	50
	Men	36	50
Patients age more than 50		39	54
Tooth type	Molar	51	63.8
	Premolar	24	30
	Incisor	5	6.3
Vital pulps^1^		63	78.8
Normal periapical diagnosis^2^		69	86.3
Fracture location (*)	M-D^3^	24	41.4
	B-L^3^	20	34.5
	Both	14	24.1
Amount of fractures (*)	Single	43	63.2
	multi	25	36.8
Presence of both adjacent teeth^4^		55	68.8
Type of restoration (*)	Amalgam	55	63
	Composite	12	14
	Full coverage	6	7
	No restoration	4	5
Extension of restoration^5^	Extensive	59	74.7
	Full coverage restoration	6	7
	Minor/none	13	16.5
Presence of caries		50	63.3
Deep periodontal pockets^6^		26	34.7

^1^Vital = the tooth was diagnosed with either normal pulp or pulpitis, Non-vital = the pulp was necrotic; ^2^categorized as ‘normal’ or ‘with periapical pathology; ^3^M-D = mesio-distal, B-L = buccolingual; ^4^present = both adjacent teeth were present; ^5^extensive restoration=the restoration covered two or more of the tooth crown aspects, but not the whole crown, minor restoration = the restoration covered one aspect of the tooth crown; ^6^deep periodontal pocket = probing depth >4 mm; ^7^In cases where the evaluated parameters were not available from the patients’ medical records, the results were presented as a percentage from the number of cases in which that parameter was available; *P < 0.05.

Fifty one (63.8%) of the cracked teeth were molar teeth (19 in the maxilla and 32 in the mandible), 24 (30%) of the cracked teeth were premolars (10 in the maxilla and 14 in the mandible), and 5 (6.3%) were anterior teeth. There were 63 (78.8%) cracked teeth with vital pulps, and 17 (21.3%) with pulp necrosis; 69 (86.3%) of the cracked teeth had a normal periapical diagnosis, while 11 (13.8%) had periapical pathology.

In 24 (41.4%) teeth the direction of the crack was M-D, in 20 (34.5%) teeth the direction of the crack was B-L, and in 14 (24.1%) teeth there were cracks in both directions. In 43 (63.2%) of the cracked teeth there was a single crack, and 25 (36.8%) teeth had multiple cracks. There was a statistically significant association between the direction of the crack and the number of cracks. Only 4% of the teeth with a M-D oriented crack had multiple cracks, while 40% of the teeth with a B-L oriented crack had multiple cracks (p < 0.05).

In 55 (68.8%) teeth both adjacent teeth were present, and in 25 (31.3%) teeth there were no adjacent teeth or one was missing. Fifty five (71.4%) of the cracked teeth had an amalgam restoration. The remaining teeth (n = 22) had either a composite restoration (n = 12, 15.6%), a full coverage restoration (n = 6, 7.8%), or no restoration (n = 4, 5.2%). Teeth restored with amalgam were at a higher risk of having multiple cracks (47%), than teeth restored with non-amalgam restorations (12%, p < 0.05).

In 59 (74.7%) teeth the extension of the coronal restoration was defined as extensive. Six (7.8%) teeth had a full coverage restoration, and in 13 (16.5%) teeth the extension of the coronal restoration was minor or the tooth had no restoration. In 50 (63.3%) teeth there were caries. There were 26 (34.7%) teeth that had deep periodontal pockets at the time of the crack diagnosis. There were no reports in the patients’ medical records on deep pockets at the time of implant placement.

The placement of an implant was found to be a risk factor for having multiple cracks. Moreover, there was a statistically significant association between the presence of an implant and the cracks’ characteristics: when there was an implant, 48% of the cracked teeth had multiple cracks in both M-D and B-L directions, while when there was no implant only 9% had multiple cracks in both M-D and B-L directions (p < 0.05). In addition, when an implant was present, there was a higher probability of having multiple cracks in both M-D and B-L directions, compared to having only a M-D oriented crack (odds ratio = 7.333, CI: 1.583 33.967, p < 0.05), only B-L oriented crack (odds ratio = 14.667, CI: 2.727 78.877, p < 0.05), or either M-D or B-L oriented crack (odds ratio = 9.777, CI: 2.320 41.216, p < 0.05).

Table 2 presents the probability of multiple cracks compared to a single crack in the presented series.

**Table 2:** The probability of having multiple cracks compared to having a single crack (P < 0.05).

Comparison vs. multiple cracks	Odds ration	95% CI
M-D oriented single crack	7.333	1.583, 33.967
B-L oriented single crack	14.667	2.727, 78.877
M-D or B-L oriented single crack	9.777	2.320, 41.216

CI = confidence interval; M-D: mesio-distal; B-L: Bucco-lingual.

## Discussion

This study reports and evaluates a series of 80 non-endodontically treated teeth diagnosed with cracks in 72 patients. In the present study strict inclusion and exclusion criteria were applied^3,15^. Only teeth diagnosed with a complete or incomplete crack initiating from the crown and extending apically, diagnosed based on a clinical and radiographic evaluation^3–5^, and presence of occlusal contacts between the cracked and the opposing tooth were included^3,12^. Tooth cracks may be divided into five sub-types: craze lines, fractured cusp, cracked tooth, split tooth and vertical root fracture (VRF)^5^. Craze lines are limited only to the tooth enamel and usually have no clinical significance other than aesthetics. Therefore, they were not included in the current study. VRFs occur primarily in endodontically treated teeth and originate in the root^13^. Therefore, they were not included in the current study as well. The terms split tooth, cracked tooth and fractured cusp, describe sub-types of tooth fractures originating in the tooth crown^5,13^. These sub-types (split tooth, cracked tooth, and fractured cusp) were categorized as one category termed “cracked teeth”, that was included in the current study^2,13^.

Based on these eligibility criteria, of a total of 285 teeth that were referred for endodontic treatment, the prevalence of cracked teeth was 28% (80/285), diagnosed in 34% (72/212) of the referred patients. Previous studies on the prevalence of cracks in non-endodontically treated teeth reported various results^16,17^. Krell *et al*.^16^ reported a prevalence rate of 9.7% of teeth with reversible pulpitis among 8,175 patients referred to a private endodontic practice over a period of six years, while Hilton and Ferracan^17^ reported that of 1,962 patients evaluated, 66.1% had at least one cracked molar. Possible reasons for these variations may be differences in the evaluated cohorts and in the methods used to diagnose and confirm the presence of the cracks. In Krell *et al*.^16^ the cracks were identified with direct transillumination and visualization, sometimes without magnification. The diagnosis of cracked teeth is not straight-forward because the symptoms are diverse, and crack lines may be difficult to identify^5^. The detection of a crack requires a valid reference standard for confirmation^15,18,19^. Therefore, in order to ensure the accuracy of the study, in the present study the method selected for verification of the cracked tooth was visual examination using magnification and illumination by a dental operating microscope and the removal of coronal restorations for observation and staining using methylene blue dye when indicated^5^.

In the current study, there was an equal distribution between men and women, with an average age of 53 years, where 39 patients (54%) were over 50 years. These characteristics of patients with cracked teeth were similar to some other studies^20,21^. However, the effect of the patient’s gender on the risk of developing a cracked tooth is a matter of controversy. In a recent study^12^, and in Cameron^22^, more than 60% of the cases were diagnosed in female patients, while in Kang *et al*.^5^, who analyzed the distribution and characteristic features of cracked teeth, cracks were more prevalent among men (61%).

It had been suggested in previous studies that older patients may be at higher risk for tooth cracks, since most cracked teeth were reported in patients over age 50^5,21–23^. A recent case series study^12^ reinforced this finding, as most patients in that study were over 50 years old (78%), and the average age was 59 years. While the exact influence of the patient’s age on the risk of cracks is not fully clarified, it has been claimed that the resistance of the tooth dentin to cracks decreases with age as a result of specific age-related biomechanical alterations such as increased stress fatigue and loss of dentin elasticity with age^5^.

In the current study, 41% of the cracked teeth were diagnosed following the placement of dental implants. The possibility that implants may be a risk factor for the development of cracks in either endodontically^3^ or in non-endodontically^12^ treated teeth was suggested only recently^3,12^. Implant supported rehabilitation has become a common practice for tooth replacement^3,24^. However, implant anchorage is different from natural teeth, as implants lack a periodontal ligament (PDL)^25^, and their anchorage is achieved by close contact between the bone and the implant (osseointegration)^26,27^. The PDL possesses unique characteristics and enables mobility, as well as stress dissemination, proprioception, and tolerance to occlusal loads, thus acting as a modulator of excessive occlusal loads. The absence of this stress modulation mechanism in osseointegrated implants may potentially expose them to occlusal overload^3,12,25^. Thus, it has been suggested that the occlusal design of implant supported rehabilitations should reduce these forces^28^.

In order to prevent excessive occlusal overloads on dental implants, the “implant-protective occlusion” principle was suggested. This principle advocates that the occlusal loads on the implant-supported prosthesis should be reduced in order to increase the odds of successful implant osseointegration^3,25,29^. Because the implant occlusion is an integral part of the patient’s occlusal system, once the occlusal design minimizes the occlusal forces applied on the dental implants, at the same time it increases the forces distributed to the patient’s natural teeth^3,30–34^. These ensuing excessive occlusal loads applied on the natural teeth expose these teeth to the risk of developing cracks^2,8,12^.

In the current study, several occlusion-related parameters were assessed, such as the number and location of the dental implants and their relation to the cracked tooth. It was found that in about a fourth of the patients (27%) the implants were located adjacent to the cracked teeth, and in half of the patients (51.5%) the implants were located at opposing or contralateral sides, or at multiple sites in the mouth (21.2%). In addition, the proportion of cracked premolars was relatively high (30% of the teeth). These results are comparable to a previous report^12^, and may be mechanistically explained by recent findings reported by Lee *et al*.^35^, that evaluated the relation between posterior dental implants and development of traumatic occlusion in the adjacent premolar teeth in 283 patients who had received 347 implants placed in the posterior region. In that cohort of patients^35^ it was found that the incidence of traumatic occlusion in the adjacent premolars increased significantly when the implants were placed in the maxillary region, in the case of splinted implants, and when implants were located in the opposing occlusion. They concluded that “the risk of traumatic occlusion in the adjacent premolars increased when splinted implants were placed in the maxillary molar region and when the teeth opposing an implant also contained implants”^35^.

An additional finding in the current study was that in most of the patients with implants, two or more implants were placed (73%), with an average of 3 implants per patient. These findings are in accordance with a recent case series study^12^, where the majority of patients with cracked teeth (78%) had multiple implants, which potentially significantly increased the loads on the natural dentition^30–34^. Therefore, it is conceivable to assume that excessive occlusal loads due to multiple implants in the molar region may contribute to the risk of cracks in adjacent non-endodontically treated premolar teeth. Additional clinical studies are warranted in order to demonstrate the exact occlusal relationship between the implants and the affected cracked teeth.

In the current study, in most teeth (70%), the time from implant loading to the diagnosis of the crack(s) was longer than 1 year, which is similar to a previous report^12^. Furthermore, most teeth (79%) presented with vital pulps and with a normal periapical diagnosis (86% of the teeth), similar to other studies^12,36^. The final diagnosis of a cracked tooth may be complicated and delayed since the tooth may be asymptomatic or with non-specific pulpal and periapical symptoms, and also as the tooth structure usually appears normal in a radiograph^22,37^. Clinically, the confirmation of the crack may be complex and requires visual examination by magnification and illumination, removal of coronal restorations for observation, and staining of the coronal tooth structure using appropriate staining materials such as methylene blue dye^5,12^.

The crack direction was distributed equally between M-D cracks and B-L cracks (41% M-D only and 34.5% B-L only), and about a fourth (24%) of the teeth exhibited cracks in both directions. These results differ from the results published by Kang *et al*.^5^ reporting that half of the teeth presented with M-D cracks, and only a few (19%) presented with B-L cracks. They have also reported that a single crack was diagnosed in only 14% of the teeth. These differences may be explained by the fact that, unlike Kang *et al*.^5^, this study evaluated only non-endodontically treated teeth, while teeth with VRFs were excluded. A recent systematic review of the literature^38^ evaluated the treatment outcome of cracked teeth that received root canal treatment and reported that the number of cracks may affect the tooth survival where teeth with a single crack exhibited a lower extraction risk^38^.

In the current study, the association between tooth cracks and placement of dental implants was also evaluated. It was found for the first time, that the placement of implants significantly increased the risk of developing multiple cracks (p < 0.05). In addition, when an implant was placed, there were significantly higher odds of having multiple cracks in both M-D and B-L directions compared to having either a M-D or a B-L oriented single crack (p < 0.05).

Interestingly, the presence of a B-L oriented crack was significantly associated with having multiple cracks, as only 4% of the teeth with a M-D oriented cracks had multiple cracks, while 40% of the teeth with a B-L oriented crack had multiple cracks (p < 0.05).

Previous investigations reported that the type and extension of a coronal restoration, as well as the presence of caries may affect the risk of developing cracks^1,2,5,22,36^. In the current study, most of the cracked teeth (71.4%) had an amalgam restoration. Furthermore, teeth restored with amalgam were at a higher risk of having multiple cracks (47%) than teeth restored with non-amalgam restorations (12%, p < 0.05). These results are in accordance with a recent study^12^, where most of the cracked teeth (61%) had an amalgam restoration. However, Kang *et al*.^5^ reported that only 6% of the cracked teeth in their cohort of cracked teeth were restored with amalgam. The difference may be partially explained by the fact that Kang *et al*.^5^ included also cases of VRFs that were excluded from both the aforementioned recent study^12^, and the current study.

The presence of multiple cracks may adversely affect the long term prognosis and survival of a cracked tooth^38,39^. Thus, it is conceivable to assume that the placement of dental implants may adversely affect the survival of cracked teeth, especially when they are restored with an amalgam restoration, by increasing the risk of multiple crack development. However, these prognostic aspects are yet to be investigated in future studies.

At the time of the crack diagnosis, about a third of the teeth (34.7%) were diagnosed with deep periodontal pockets that were not diagnosed previously. It had been claimed that deep probing depth may adversely affect the prognosis of cracked teeth^1,5^. Leong *et al*.^38^ reported in a recent systematic review of the literature that cracked teeth with periodontal pockets >3 mm are at a greater risk of extraction following root canal treatment^38^. Kang *et al*.^5^ classified probing depths by 3-mm intervals, and reported that about half of the teeth had probing depths of 3 or more mm, and that probing depths of more than 6 mm significantly reduces the survival of cracked teeth treated with a root canal treatment. Kim *et al*.^1^ also reported that the tooth prognosis was less favorable in cracked teeth with a deep probing depth. Abulhamael *et al*. reported in a recent survey of American endodontists^40^ that the presence of a deep periodontal pocket is considered an important factor by most American endodontists when deciding whether to preserve the cracked tooth or extract it^40^.

## Conclusions

Based on strict inclusion criteria and confirmation methods, the prevalence of cracked non-endodontically treated teeth in patients referred for endodontic treatment is high, reaching as much as a third of the cases. Almost half of the cracked teeth were diagnosed following the placement of dental implants, and almost a third of the cases may be diagnosed in premolars.

Implant placement, and the presence of amalgam restorations may increase the risk of multiple cracks, and thus potentially adversely affecting the survival of the cracked teeth. Clinicians should be aware of the common patient profile found in this study, that being a patient having a non-endodontically treated premolar restored with an amalgam restoration and which was diagnosed with multiple cracks more than 1 year after reconstruction based on multiple implants.

Additional prospective studies with larger samples are warranted in order to better understand the possible correlations between implant rehabilitation and cracked teeth, as well as to understand the effect of these ensuing cracks on the survival of these teeth.

## Data Availability

The datasets generated during and/or analysed during the current study are available from the corresponding author on reasonable request.
